# Supplementary material for: End Sequence Analysis Toolkit (ESAT) expands the extractable information from single-cell RNA-seq data
Source: Genome Res. 2016 Oct;26(10):1397–410. doi: 10.1101/gr.207902.116 (PMC5052061; doi:10.1101/gr.207902.116)
Supplement: Supplemental Material [file supp_gr.207902.116_Supp_Materials.tar.gz › ESAT-esat_v0.1/doc/html/functions_func_b.html]

ESAT: Class Members - Functions


ESAT


- Main Page
- Related Pages
- Packages
- Classes
- Files

- Class List
- Class Index
- Class Hierarchy
- Class Members

- All
- Functions
- Variables
- Enumerator

- a
- b
- c
- d
- e
- f
- g
- h
- i
- j
- k
- l
- m
- n
- o
- p
- q
- r
- s
- t
- u
- v
- w
- z


All Classes Namespaces Files Functions Variables Enumerator Pages

### - b -

- BadModelException()
  : broad.core.hmm.BadModelException
- BasicAnnotation()
  : umms.core.annotation.BasicAnnotation
- BasicAnnotationReader()
  : broad.core.annotation.BasicAnnotationReader
- BasicGenomicAnnotation()
  : broad.core.annotation.BasicGenomicAnnotation
- BasicGenomicAnnotationReader()
  : broad.core.annotation.BasicGenomicAnnotationReader
- BasicLightweightAnnotation()
  : broad.core.annotation.BasicLightweightAnnotation
- BasicScanStatisticScore()
  : umms.core.model.score.BasicScanStatisticScore
- BasicTwoSubjectAnnotation()
  : broad.core.annotation.BasicTwoSubjectAnnotation
- BatchNorm()
  : broad.pda.differentialExpression.BatchNorm
- BED()
  : broad.core.annotation.BED
- bed2CuffGtf()
  : broad.pda.annotation.BEDFileParser
- bed2gtf()
  : broad.pda.annotation.BEDFileParser
- bed2gtf\_bundleOverlappers()
  : broad.pda.annotation.BEDFileParser
- BEDFileParser()
  : broad.pda.annotation.BEDFileParser
- BEDGraph()
  : broad.core.annotation.BEDGraph
- BEDGraphReader()
  : broad.core.annotation.BEDGraphReader
- BEDReader()
  : broad.core.annotation.BEDReader
- begin()
  : broad.core.annotation.AnnotationHandler
  , broad.pda.datastructures.LocationAwareMatrix
- binomialCoefficient()
  : broad.core.math.MathUtil
- BinomialEnrichmentScore()
  : umms.core.model.score.BinomialEnrichmentScore
- breakUpMultifile()
  : broad.core.sequence.FastaSequenceIO
- browserLine()
  : broad.core.annotation.AnnotationHandler
  , broad.pda.datastructures.LocationAwareMatrix
- buildKmerMap()
  : broad.core.sequence.Sequence
- BuildScriptureCoordinateSpace()
  : umms.core.scripture.BuildScriptureCoordinateSpace


---

Generated on Fri Aug 15 2014 15:11:16 for ESAT by  

 1.8.7
